# Making a case for the combined use of SGLT2 inhibitors and GLP1 receptor agonists for cardiorenal protection

**DOI:** 10.1590/2175-8239-JBN-2020-0100

**Published:** 2020-09-11

**Authors:** Vikas S. Sridhar, Lisa Dubrofsky, Jacinthe Boulet, David Z. Cherney

**Affiliations:** 1 University of Toronto, Department of Medicine, Division of Nephrology, Toronto General Hospital, Toronto, Ontario, Canada.; 2 University of Montreal, Department of Medicine, Division of Cardiology, Montreal, Quebec, Canada.; 3 University of Toronto, Banting and Best Diabetes Centre, Toronto, Ontario, Canada.; 4 University of Toronto, Departments of Physiology and Pharmacology and Toxicology, Toronto, Ontario, Canada.

**Keywords:** Sodium-Glucose Transporter 2 Inhibitors, Diabetes Mellitus, Type 2, Glucagon-Like Peptide-1 Receptor, Diabetic Nephropathies, Cardiovascular Diseases, Heart Failure, Inibidores do Transportador 2 de Sódio-Glicose, Diabetes Mellitus Tipo 2, Receptor do Peptídeo Semelhante ao Glucagon 1, Nefropatias Diabéticas, Doenças Cardiovasculares, Insuficiência Cardíaca

## Abstract

Sodium glucose cotransporter-2 (SGLT2) inhibitors and glucagon-like peptide-1 receptor agonists (GLP-1RA) were initially approved to improve glycemic control in the treatment of type 2 diabetes. Clinical trials have also demonstrated beneficial effects with regards to cardiovascular and renal parameters. Beyond improving glycemic control, these therapies promote weight loss and lower blood pressure when used individually, and in an additive manner when used together. Accordingly, taking advantage of complementary mechanisms of action with the combined use of these two classes of agents to further improve cardiorenal outcomes is conceptually appealing, but has yet to be explored in detail in clinical trials. In this review, we discuss proposed mechanisms for renal protection, clinical benefits, and adverse events associated with the individual and combined use of SGLT2 inhibitors and GLP-1RA. The management of type 2 diabetes has significantly changed over the last few years, moving away from solely glycemic control towards the concurrent management of associated comorbidities in a patient population at significant risk of cardiovascular disease and progression of chronic kidney disease. It is from this perspective that we seek to outline the rationale for the sequential and/or combined use of SGLT2 inhibitors and GLP-1RA in patients with type 2 diabetes.

## RESUMO

Inibidores do cotransporter-2 de glicose sódica (SGLT2) e agonistas do receptor peptídeo-1 do tipo glucagon (GLP-1RA) foram inicialmente aprovados para melhorar o controle glicêmico no tratamento da diabetes tipo 2. Os ensaios clínicos também demonstraram efeitos benéficos em relação aos parâmetros cardiovasculares e renais. Além de melhorar o controle glicêmico, essas terapias promovem perda de peso e redução da pressão arterial quando usadas individualmente, e de forma aditiva quando usadas em conjunto. Consequentemente, tirar proveito de mecanismos de ação complementares com o uso combinado dessas duas classes de agentes para melhorar ainda mais os resultados cardiorrenais é conceitualmente atraente, mas ainda precisa ser explorado em detalhes em ensaios clínicos. Nesta revisão, discutimos os mecanismos propostos para proteção renal, benefícios clínicos e eventos adversos associados ao uso individual e combinado de inibidores de SGLT2 e GLP-1RA. O tratamento do diabetes tipo 2 mudou significativamente nos últimos anos, passando do controle exclusivamente glicêmico para o tratamento simultâneo de comorbidades associadas em uma população de pacientes com risco significativo de doença cardiovascular e progressão da doença renal crônica. É nessa perspectiva que procuramos delinear a justificativa para o uso sequencial e/ou combinado de inibidores de SGLT2 e GLP-1RA em pacientes com diabetes tipo 2.

## INTRODUCTION

Based on the results of recent cardiovascular outcome trials (CVOT) using newer glucose lowering therapies, diabetes clinical practice guidelines have had a renewed focus on cardiorenal protection in patients at significant risk for cardiovascular (CV) and kidney disease. In contrast to other anti-hyperglycemic agents, sodium-glucose contransport-2 (SGLT2) inhibitors and glucagon-like peptide-1 receptor agonists (GLP-1RA) have individually demonstrated CV and kidney benefits, as described below. Moreover, combined use of these medication classes in patients with T2D is appealing due to possible mechanistic and clinical synergies. In this article, we review the mechanisms and clinical benefits of SGLT2 inhibitors and GLP-1RA and outline the rationale for their sequential or combined use in people with type 2 diabetes (T2D).

## PROPOSED MECHANISMS FOR RENAL PROTECTION

### SGLT2 INHIBITORS

It is important to have a general overview of the main hypotheses that have been put forward to explain cardiorenal protection with SGLT2 inhibitors. Detailed reviews of mechanisms linked with cardiorenal protection have been published elsewhere.^1^^-^ 3 Indirect mechanisms - those not directly attributable to intrinsic renal pathways - that may be protective include: 1) Improved glycemic control with improved insulin sensitivity; 2) Body weight loss; and 3) Blood pressure (BP) lowering possibly via decreases in arterial stiffness and modulation of neurohormonal pathways^4^^-^ 7. There are also several proposed direct renal effects including^2^^,^ 3 ^,^ 8: 1) Reduced intraglomerular pressure by activating tubuloglomerular feedback (TGF) on the basis of acute proximal tubular natriuresis^9^^-^ 12 leading to afferent arteriolar vasoconstriction, particularly in the setting of renal hyperfiltration^13^^-^ 19; 2) Suppression of pro-inflammatory and pro-fibrotic factors in the kidney, possibly via reducing hyperglycemia and by suppressing the renin-angiotensin-aldosterone system (RAAS)^20^^-^ 24 and also by reducing intraglomerular pressure; and 3) Reducing renal ischemia via multiple pathways, thereby reducing kidney injury^25^^,^ 26. While it is not known which of these hypotheses is most closely related to kidney protection, the largest body of experimental and human trial evidence exists for changes in glomerular hypertension, which could also explain the rapid anti-proteinuric effect achieved with these agents and the rapid, clinically relevant, reversible “dip” in eGFR seen with these agents^27^^,^ 28.

### GLP-1 RECEPTOR AGONISTS

GLP-1RA may similarly have “indirect” and “direct” effects contributing to their kidney protection. For indirect mechanisms, GLP-1RA therapies have a variety of effects on metabolism that may be cardiorenal protective, including reduced postprandial glucose levels by stimulating insulin secretion, reducing glucagon release and hepatic gluconeogenesis, and slowing gastric emptying^29^. GLP-1RA additionally act at the central nervous system level, increasing satiety leading to weight loss^30^^-^ 32. GLP-1RA may also lower blood pressure through effects on the endothelium, and increase heart rate (HR) through stimulation of the sinoatrial node and the sympathetic nervous system (SNS)^33^^,^ 34. These agents also appear to have anti-atherosclerotic effects through several mechanisms including anti-inflammatory effects at the level of the vessel wall and reduced endothelial dysfunction^29^^,^ 35, which could also protect the kidney. Proposed direct renal beneficial effects include: 1) natriuresis contributing to BP reduction; 2) albuminuria reduction, possibly mediated by suppression of inflammatory-related pathways; and 3) reduction in renal inflammation and oxidative stress related mechanisms^36^^-^ 38. Pathways leading to GLP-1RA induced natriuresis are unclear, with inhibition of the sodium hydrogen exchanger-3 (NHE3) antiporter in the proximal tubule being one possibility^39^^-^ 41. Despite this proximal natriuresis, unlike with SGLT2 inhibition, blockade of sodium reabsorption at this level of the nephron with GLP-1RA does not lead to a characteristic and reversible dip in eGFR with these therapies. Even though glomerular hemodynamics remain unaffected with GLP-1RA, these therapies are linked with a rapid reduction in albuminuria, possibly on the basis of direct anti-inflammatory GLP-1 pathways that may not be dependent on natriuresis^39^^,^ 42.

## IMPACT OF SGLT2 INHIBITORS AND GLP-1RA ON GLYCEMIC CONTROL, BLOOD PRESSURE, AND WEIGHT

GLP-1RAs and SGLT2 inhibitors have beneficial clinical effects on glycemic control, blood pressure, and weight loss, when used alone or together. In terms of glycemic effects, GLP-1RAs reduce mean HbA1c by 0.55-1.2% versus placebo in patients with T2D, with exaggerated effects seen with longer-acting agents^43^. The glucose-lowering effects of SGLT2 inhibitors are achieved via glucosuria, and are comparatively modest, reducing HbA1c by 0.5-0.7% in patients with normal kidney function, and lesser effects are seen in patients with CKD stage 3 or 4^2^^,^ 44. Both agents also have systemic hemodynamic effects. GLP-1RA lower systolic and diastolic BP to varying degrees, with estimates of 1.2- 4.6 mmHg systolic / 0 - 1.1 mmHg diastolic^45^^,^ 46. One study using 24-hour ambulatory blood pressure monitoring over 5 weeks reported a more robust systolic BP lowering effect of 5 mmHg with liraglutide, with a neutral effect on diastolic BP^47^. The effects on BP are independent of glucose control and weight loss^33^. BP lowering with GLP-1RA is associated with increases in HR by 2-3 beats/minute in large clinical trials and up to 6-10 beats/minute in 24-hour monitoring studies^34^^,^ 45. SGLT2 inhibitors have a mean BP lowering effect of 3.8 mmHg systolic/1.4 mmHg diastolic compared to placebo, an effect which appears to be largely independent of the baseline BP and HbA1c lowering, and preserved in patients with CKD^2^^,^ 4 ^,^ 7. SGLT2 inhibitors do not affect heart rate^6^^,^ 48. Finally, both agents promote weight loss. GLP-1RA induce weight loss ranging from 0.2-7.2 kg, with approximately 50% of patients achieving a weight loss of ≥5% on therapy^49^. SGLT2 inhibitors lead to a modest weight loss of 2-3 kg, including both fluid and adipose tissue loss. The magnitude of SGLT2 inhibitor-induced weight loss is largely preserved in patients with CKD^4^^,^ 44.

Based on their distinct mechanisms of action and clinical effects, GLP-1RAs and SGLT2 inhibitors may have complementary renal and systemic effects, leading to interest in the potential for their combined use. There is, however, limited data on the combined use of GLP-1RAs plus SGLT2 inhibition on improving glycemic control, blood pressure, and weight. In the 28-week, phase three randomized control trial (RCT) DURATION-8, dual therapy with an SGLT2 inhibitor (dapagliflozin) and a GLP-1RA (once-weekly exenatide) was shown to reduce HbA1c by 0.4% more than either agent alone, in patients with a starting HbA1c of between 8-12%^50^, illustrating that the glycemic response to dual therapy is less than additive^50^. At the conclusion of DURATION-8, systolic BP was reduced by 1.3 mmHg for patients on single-agent exenatide, 1.8 mmHg for those on single-agent dapagliflozin, and 4.2 mmHg with combined therapy, supporting an additive BP lowering effect of combined therapy^50^. DURATION-8 demonstrated an additive effect of dual therapy in inducing weight loss, with an additional 1.87 kg and 1.20 kg of weight loss versus single-agent exenatide and dapagliflozin, respectively^50^.

Further data comes from AWARD-10, a 24-week, phase three RCT of placebo versus add-on therapy with the GLP-1RA dulaglutide in patients on baseline SGLT2 inhibition^51^, and SUSTAIN-9, a 30-week trial looking at add-on therapy with the GLP-1RA semaglutide to baseline SGLT2 inhibition^52^. Both trials included patients with baseline HbA1c lower limit of 7% (mean HbA1c of 8%)^51^^,^ 52. Add-on therapy with dulaglutide 1.5 mg weekly (but not 0.75 mg) to baseline SGLT2 inhibition in AWARD-10 resulted in 0.9 kg additional weight loss compared to placebo, while add-on therapy with semaglutide 1 mg weekly in SUSTAIN-9 resulted in an additional 3.1 kg weight loss versus placebo^51^^,^ 52. A separate trial examined dual therapy with dapagliflozin and exenatide in 50 patients with obesity but without T2D in a single-center RCT^53^. At 24 weeks, patients in the active arm achieved 4.1-kg more weight loss than placebo, with 36% (versus 4.2% in placebo) achieving ≥5% body weight loss^53^.

Despite what is known about HbA1c, weight, and blood pressure effects of combination therapy, less is known about other mechanisms associated with cardiorenal protection in response to combination therapy such as natriuresis and renal hemodynamics. In the following sections, we describe individual cardiorenal benefits of these agents before outlining a proposal for their combined used.

## CARDIORENAL BENEFITS OF SGLT2 INHIBITORS AND GLP-1 RECEPTOR AGONISTS FROM RENAL AND CARDIOVASCULAR OUTCOME TRIALS

Among large-scale clinical trials involving SGLT2 inhibitors and GLP-1RA, there exists only one dedicated renal outcome trial that has been published to date. The CREDENCE trial involved patients with T2D, macroalbuminuria, and eGFR 30-90 mL/min/1.73m^2^.^54^ In this trial involving the SGLT2 inhibitor canagliflozin versus placebo, the risk of the primary outcome - doubling of serum creatinine, end-stage kidney disease, renal death, or CV death - was reduced by 30% with canagliflozin. More recently, the DAPA-CKD study (NCT03036150), studying renal outcomes with dapagliflozin in proteinuric CKD patients (≥25 and ≤75 mL/min/1.73m^2^) with or without diabetes was stopped early due to overwhelming efficacy with dapagliflozin. The EMPA-KIDNEY trial (NCT03594110) is similarly studying the effect of empagliflozin in patients with diabetic chronic kidney disease (DKD) or non-diabetic chronic kidney disease (CKD), and has a lower limit of inclusion, GFR at ≥20 mL/min/1.73m^2^. Until the results of these studies are available, CVOTs and CREDENCE account for the current dataset related to renal protection across a large cohort of patients with a range of baseline kidney and CV disease related to T2D.

### SGLT2 INHIBITORS

Prior to CREDENCE, three CVOTs evaluated SGLT2 inhibitors in participant cohorts with varying degrees of CV and renal risk. EMPA-REG OUTCOME included 7020 patients with established atherosclerotic CV disease (ASCVD)^48^, while the CANVAS Program trial included 10,142 with two-thirds having ASCVD^55^. The most recent DECLARE TIMI-58 had 17,160 patients with approximately 40% having ASCVD^56^. In addition, the trial cohorts also had differences in baseline renal health, with the highest mean baseline eGFR in DECLARE TIMI-58.

EMPA-REG OUTCOME first demonstrated significant reductions in primary 3-point MACE endpoint, CV mortality, overall mortality, and hospitalization for heart failure (HHF) in its entire cohort and in subgroups with varying baseline renal function and proteinuria^48^^,^ 57. In renal endpoint analyses, Wanner et al. reported a 39% risk reduction for the renal composite of albuminuria progression, serum creatinine doubling or end-stage kidney disease (ESKD), or renal death^58^. Importantly, each component - aside from renal death - was reduced and renal benefits were consistent across multiple subgroups including those with CKD at baseline^58^. Beyond effects on “hard” renal endpoints, empagliflozin also resulted in a reversible eGFR “dip” after approximately 4 weeks of therapy. After 4 weeks, eGFR was better preserved compared to placebo, an effect that was greatest in patients with baseline macroalbuminuria, where placebo-treated patients lost ≈20 mL/min/1.73m^2^ over 3.1 years compared to ≈10 mL/min/1.73m^2^ in the empagliflozin-treated arm^28^. Interestingly, albuminuria reductions in EMPA-REG OUTCOME persisted in participants with baseline albuminuria even after drug washout, suggesting renal structural changes beyond simple hemodynamic effects^28^. Finally, renal benefits were independent of HbA1c lowering, while hematocrit/hemoglobin - as markers of hemoconcentration with natriuresis - were most closely associated with CV protection^59^^,^ 60. This supports the hypothesis that protection against diabetes-related complications are independent of metabolic improvements with SGLT2 inhibitors.

Subsequent to the EMPA-REG OUTCOME trial being published, the CANVAS Program reported its results. This trial comprised an intermediate CV risk cohort and also demonstrated significant reductions in MACE, without individual improvements in all-cause or CV mortality^55^^,^ 61 ^,^ 62. Foreshadowing CREDENCE, canagliflozin significantly reduced the composite of sustained doubling of serum creatinine, ESKD or renal death, reduced annual eGFR decline by 1.2 mL/min/1.73 m^2^/year, and UACR decreased by 18% in the overall cohort^63^. Two years later, DECLARE-TIMI 58 was completed, and reported the impact of SGLT2 inhibition with dapagliflozin in the lowest CV risk cohort^56^. Aside from a 27% reduction in the co-primary endpoint of CV death or HHF (largely driven by HHF reduction), dapagliflozin reduced the risk of 40% decline in eGFR to <60 mL/min/1.73m^2^, ESKD or renal death by 47%^64^. Hazard ratios for CV outcomes and mortality were generally similar across baseline levels of eGFR and albuminuria, aside from a lower HR in the impaired eGFR with albuminuria group^65^. These results demonstrate a consistent role for cardiorenal protection with SGLT2 inhibitors in relatively low renal risk population presumably early in their disease course.

The benefit of SGLT2 inhibition on HHF was further shown in the DAPA-HF trial, which importantly included participants with heart failure and reduced ejection fraction (HFrEF) with and without T2D^66^. Dapagliflozin was effective in reducing the risk of the primary outcome - composite of worsening heart failure, cardiovascular death, or urgent care visit for heart failure - in participants with and without T2D. Benefits on the primary composite endpoint were present across major baseline groups of clinical characteristics, including use of background heart failure therapies.^67^ In DAPA-HF, dapagliflozin was well-tolerated, even in elderly trial participants.^68^ In secondary analyses, there was a trend towards reducing the risk of the composite renal outcome (≥50% sustained decline in eGFR/ESKD/renal death - HR 0.71, 95% CI 0.44-1.16), with the caveat that events rates were low. The investigators also reported a significant separation in the eGFR slope curves favoring dapagliflozin, even when stratified by T2D status.

### GLP-1 RECEPTOR AGONISTS

To date, seven CVOTs assessing GLP-1RA have been published, combining a total of 56,004 patients. Multiple meta-analyses certainly confirm a benefit in terms of reductions in MACE in patients with established ASCVD, and reductions in the risk of stroke^69^^-^ 71. The benefits of GLP-1RA in those without established ASCVD and in reducing the risk of HHF is less clear, and individual trials have not yet demonstrated protection against HHF^71^.

For kidney protection, benefits have been reported with GLP-1RA, though less substantial compared to those that have been established in the SGLT2 inhibitor literature. In pre-specified secondary renal outcome analyses in LEADER, new-onset persistent macroalbuminuria, persistent doubling of serum creatinine, need for renal replacement therapy (RRT), and death due to renal disease were reduced with a hazard ratio of 0.78 and an NNT of 67 (95% CI 0.67-0.92; P=0.003)^46^^,^ 72. This was, however, driven by a reduction in new onset persistent macroalbuminuria, though the number of ‘hard’ renal outcomes was low - 120 of 9340 patients required RRT, 184 of 9430 had a doubling of serum creatinine. In a similar population, SUSTAIN-6 compared semaglutide with placebo and demonstrated a reduction in new or worsening nephropathy with a hazard ratio of 0.64 (95% CI 0.46-0.88; P=0.005)^73^, again an effect based on a lower risk of albuminuria progression. In an exploratory renal outcome analysis of REWIND, dulaglutide reduced the composite outcome of first occurrence of new macroalbuminuria (UACR >33.9 mg/mmol), a sustained decline in eGFR of 30% or more from baseline, or chronic RRT (HR 0.85, 95% CI 0.77-0.93; p=0.0004) - also driven by a reduction in new onset macroalbuminuria^74^^,^ 75. Meta-analyses of GLP-1RA agents have confirmed that relative risk reductions in composite renal outcomes are largely driven by reductions in macroalbuminuria^69^^-^ 71.

Perhaps the most convincing data for kidney protection beyond albuminuria lowering stems from the AWARD-7 trial. This study evaluated dulaglutide against insulin glargine in 577 patients with moderate-to-severe CKD (stages 3-4, with an approximate mean eGFR of 38 mL/min/1.73m^2^) for the primary outcome of HbA1c reduction at 26 weeks and secondary outcomes of eGFR and UACR^76^. By design, glycemic lowering between the two treatment arms was similar. Despite glycemic equipoise, dulaglutide was associated with a smaller decline in eGFR at one year, particularly in patients with baseline macroalbuminuria, who also achieved the greatest reduction in urinary albumin-to-creatinine ratio with the higher 1.5 mg dulaglutide dose. This important observation emphasizes that, similar to renal benefits achieved with SGLT2 inhibitors, GLP-1RA effects in the kidney are likely glucose-independent.

## HOW TO USE THESE AGENTS TOGETHER?

**Figure 1:**
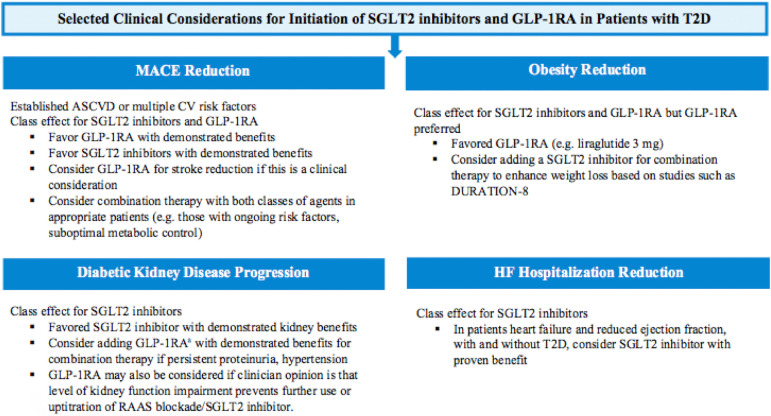
T2D management with SGLT2 inhibitors and GLP-1 RA. _a_Caveat that strong evidence for reduction in hard renal outcomes with GLP1-RA is limited.

As observed in trials such as DURATION-8, when used together, SGLT2 inhibitors and GLP-1RA confer additional benefits on glycemic control, BP, and weight loss. However, there are currently no completed clinical trials studying impact of combined SGLT2 inhibitor plus GLP-1RA on CV use on renal outcomes. A *post-hoc* propensity-matched cohort from the EXSCEL trial with the GLP-1RA exenatide demonstrated that the addition of exenatide in patients on a background of SGLT2 inhibition tended to exhibit improvements in MACE and all-cause mortality, and slower rates of renal decline compared to patients not on an SGLT2 inhibitor^77^. In real-world practice, SGLT2 inhibitors and GLP-1RA are likely to be initiated sequentially. In line with multiple clinical practice guidelines, we recommend a patient-centered approach targeting diabetes-related cardiorenal comorbidities and risk factors, while considering the risk of adverse events, patient preference, and cost (Figure 1).

In obese patients with T2D without other comorbidities, while both agents promote weight loss, GLP-1RA may be preferred as the first agent, due to greater weight loss at the upper range than SGLT2 inhibitors, particularly with the longer acting liraglutide and semaglutide^78^. Additionally, weight loss with SGLT2 inhibitors may be limited by compensatory adaptations including hyperphagia^79^. Indeed, liraglutide 3 mg daily is approved as a weight loss agent when used as an adjunct to a reduced caloric diet and physical activity. Given the significant heterogeneity in weight loss with GLP-1RA, inadequate responses may prompt the addition of SGLT2 inhibition in light of results of DURATION-8 and AWARD-10.

Among patients with T2D and established ASCVD, both GLP-1RA and SGLT2 inhibitors reduce MACE by similar magnitudes and initiation of either class is indicated. Such patients may subsequently be prime candidates for combination therapy. In patients with T2D and multiple risk factors for ASCVD but without established disease, meta-analyses have not detected a MACE benefit for GLP-1RA or SGLT2 inhibitors.^69^ Other considerations may include the type of background ASCVD. For example, semaglutide and dulaglutide have demonstrated reductions in risk of stroke, and similar effects have been reported in the class as a whole in meta-analyses^70^^,^ 73 ^,^ 75. Accordingly, it may be reasonable to preferentially initiate patients with T2D and a history of stroke on a GLP-1RA as the first agent prior to using an SGLT2 inhibitor.

In patients with T2D and heart failure, especially HFrEF, SGLT2 inhibition should be prioritized in addition to other guideline-directed therapy, as demonstrated in the DAPA-HF trial^66^. Additionally, multiple meta-analyses have demonstrated reductions in HHF with SGLT2 inhibitors, reductions that have not been demonstrated with GLP-1RA^69^. Moreover, results of the FIGHT study, where patients with HFrEF were randomized to liraglutide vs placebo, suggested a trend towards harm^80^. One possible reason for this observation could be the opposing chronotropic effects of SGLT2 inhibitors and GLP-1RA discussed in the sections above. The chronotropic effects of GLP-1RA may be particularly deleterious in heart failure patients with higher sympathetic activity contributing to morbidity and mortality^34^^,^ 80. Conversely, SGLT2 inhibitors are not associated with increased HR despite natriuresis/osmotic diuresis and reduced blood pressure and plasma volume, suggestive of reduced SNS activity^6^^,^ 81. The addition of GLP-1RA to SGLT2 inhibitors in HF patients with T2D might be considered with a concomitant history of ASCVD. It remains to be seen whether SGLT2 inhibitors diminish increases in HR when used in combination with GLP-1RA.

Finally, in T2D patients with DKD, particularly those with high risk of progression, results from CREDENCE and from secondary CVOT analyses strongly support the use of SGLT2 inhibitors. Anticipated results of the DAPA-CKD and from EMPA-Kidney may also make a case for the use of SGLT2 inhibitors in non-diabetic proteinuric CKD. Persistent proteinuria and/or hypertension, despite maximal dose RAAS blockade plus SGLT2 inhibitor use, may prompt consideration of adding a GLP-1RA. Importantly, the benefits of SGLT2 inhibition have largely been demonstrated in study cohorts already taking background treatments that are cardiorenal protective, such as RAAS blockade. RAAS blockade and SGLT2 inhibitors are both associated with significant renal hemodynamic mediated dips in GFR, which may limit their use in patients with renal function at lower limits of clinical trial inclusion criteria (≤30 mL/min in the CREDENCE trial or ≤25 mL/min in the DAPA-CKD trial). The addition of eGFR neutral GLP-1RA to hemodynamically active RAAS inhibition or SGLT2 inhibition may be desirable under such circumstances given their possible direct renal benefits that are independent of acute GFR “dip” effects.

## ADVERSE EVENTS

SGLT2 inhibitors are well tolerated, with mycotic genital infections the most common side effect and diabetic ketoacidosis, a rare but potentially serious complication^82^. Reports of acute kidney injury (AKI) have not been substantiated, with all three CVOTs and CREDENCE reporting reductions in AKI^83^. An increased risk of lower extremity amputation was observed in CANVAS and a few real-world studies, but not in CREDENCE^54^^,^ 55 ^,^ 84, suggesting that this is no longer a major concern. GLP-1RA have been associated with dose-dependent gastrointestinal effects including nausea, vomiting, and diarrhea in approximately 5-10% of people^85^. Animal studies and observational data on risks of pancreatitis and medullar thyroid cancer have not been supported in CVOTs^86^^,^ 87. More recent *post-hoc* analyses have shown that GLP-1RA is safe and well tolerated in patients with kidney disease^88^^,^ 89.

**Figure 2:**
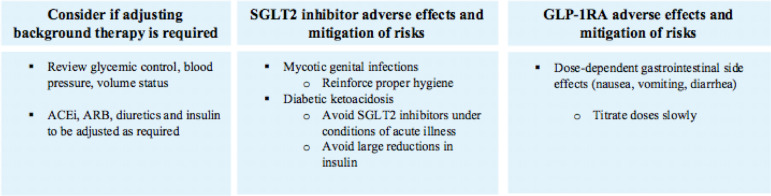
Monitoring and side effect considerations.

GLP-1RA have a glucose-dependent mechanism of action and low rates of hypoglycemia^90^. The risk of hypoglycemia is similarly low with SGLT2 inhibition because the effects on urinary glucose excretion are dampened at lower glucose levels^2^. Importantly, there was no increased risk of hypoglycemia seen with dual therapy in DURATION-8, and minimal risk in AWARD-10 and SUSTAIN-9^50^^-^ 52. Overall, these trials suggest an acceptable safety profile with combined therapy and no specific signal for heightened risk of adverse effects (Figure 2).

## CONCLUSIONS

The use of SGLT2 inhibitors and GLP1-RA individually has significantly increased over the past several years. With the emergence of clinical trials in non-diabetic patient populations demonstrating benefits of these agents in cardiovascular and renal outcomes, use of these drugs is anticipated to expand into the arsenal of nephrologists, cardiologists, and neurologists. We expect their combined use to similarly increase, particularly in patients with multiple comorbidities. With respect to their individual use, important questions remain unanswered, including whether or not GLP-1RA reduces the risk of end stage kidney disease or doubling of serum creatinine in cohorts with DKD at baseline - a question currently being studied in the ongoing FLOW trial with semaglutide. In addition, it is not known if there is a lower kidney function limit for cardiorenal protection with either class of drug, especially in patients with non-diabetic conditions. Finally, patients with type 1 diabetes and solid organ transplant recipients, particularly kidney transplant recipients, are other populations in which ongoing investigation for use of these agents is warranted. Ultimately, based on data from studies using individual therapies, it is now of the utmost importance to elucidate the role of combination SGLT2 inhibitor-GLP-1RA therapy as a strategy to reduce cardiorenal complications.
